# Evolutionary and structural analyses of the NADPH oxidase family in eukaryotes reveal an initial calcium dependency

**DOI:** 10.1016/j.redox.2022.102436

**Published:** 2022-08-12

**Authors:** Marta Massari, Callum R. Nicoll, Sara Marchese, Andrea Mattevi, Maria Laura Mascotti

**Affiliations:** a Department of Biology and Biotechnology Lazzaro Spallanzani, University of Pavia, Via Ferrata 9, 27100, Pavia, Italy; b Molecular Enzymology, Groningen Biomolecular Sciences and Biotechnology Institute, University of Groningen, Nijenborgh 4, 9747, AG Groningen, the Netherlands; c IMIBIO-SL CONICET, Facultad de Química Bioquímica y Farmacia, Universidad Nacional de San Luis, Ejercito de los Andes 950, D5700HHW, San Luis, Argentina

**Keywords:** NADPH oxidase, NOX, Reactive oxygen species, Enzyme evolution, NOX, NADPH oxidase, ROS, reactive oxygen species, DUOX, dual oxidase, RBOH, respiratory burst oxidase, MSA, multiple sequence alignment, ML, Maximum Likelihood, TBE, Transfer Bootstrap Expectation, ASR, Ancestral Sequence Reconstruction

## Abstract

Reactive oxygen species are unstable molecules generated by the partial reduction of dioxygen. NADPH oxidases are a ubiquitous family of enzymes devoted to ROS production. They fuel an array of physiological roles in different species and are chemically demanding enzymes requiring FAD, NADPH and heme prosthetic groups in addition to either calcium or a various number of cytosolic mediators for activity. These activating partners are exclusive components that partition and distinguish the NOX members from one another. To gain insight into the evolution of these activating mechanisms, and in general in their evolutionary history, we conducted an in-depth phylogenetic analysis of the NADPH oxidase family in eukaryotes. We show that all characterized NOXs share a common ancestor, which comprised a fully formed catalytic unit. Regarding the activation mode, we identified calcium-dependency as the earliest form of NOX regulation. The protein-protein mode of regulation would have evolved more recently by gene-duplication with the concomitant loss of the EF-hands motif region. These more recent events generated the diversely activated NOX systems as observed in extant animals and fungi.

## 1 Introduction

NADPH oxidases (NOXs; E.C. 1.6.3.1) are a family of transmembrane enzymes that produce reactive oxygen species (ROS; [[1], [2], [3]]) as sole product, through the oxidation of NADPH. They belong to the ferric reductase superfamily, named after its characteristic heme-containing transmembrane domain [4]. NOX enzymes share a conserved catalytic core featuring six transmembrane α-helices that encapsulate two heme-binding sites, known as the transmembrane domain, and a C-terminal cytosolic dehydrogenase domain comprised of NADPH and FAD binding sites. NOXs first oxidize a molecule of NADPH in the dehydrogenase domain using the FAD cofactor. The two electrons are then transferred across the membrane through the two heme prosthetic groups. The final electron acceptor, O_2_, is reduced by the outer heme, producing O_2_^−●^ or H_2_O_2_ [3,[5], [6], [7]]. ROS generation thereby usually occurs on the outer side of the cell membranes, a most important functional feature of NOXs. However, NOX-mediated ROS production can also occur in the cytosol. For instance, some NOX homologs (NOX1, NOX4 and NOX5) have been found to localize in the endoplasmic reticulum, where the ROS produced is involved in intracellular signaling [[5], [6], [7], [8]]. Moreover, in phagocytes, superoxide production by NOX2 occurs inside the phagosomes to kill pathogens.

Over the last couple of decades, NOXs have gained significant attention regarding their roles in cellular function. To date, many family members have been characterized and are prevalent across eukaryotic species. However, some prokaryotic sequences have been identified, sharing the same catalytic core of eukaryotic sequences. Most of them are constitutively active as single polypeptide chains and are evolutionary distant from their eukaryotic counterparts (*e.g.* the *Streptococcus pneumoniae* NOX [9]). The very few well-identified cyanobacterial NOX5 sequences are a special case as they are likely the result of a horizontal gene transfer event from a eukaryotic donor [4,9]. Our analysis will therefore focus on the eukaryotic NOXs.

Mammals encode seven NOXs including NOX1, NOX2, NOX3, NOX4, NOX5, and the Dual Oxidases (DUOX) 1 and 2 that possess an additional peroxidase-like domain. Fungal organisms harbour three main homologs called NOXA, NOXB and NOXC, whilst plants have several paralogs denoted as respiratory burst oxidases (RBOHs). To date, several of their physiological functions include, but are not limited to, cell signalling, phagocyte function in innate immunity (NOX2), host defence (NOXA, RBOHs and NOX2), thyroid hormone synthesis (DUOXs), tolerance to stress and plant cell signaling (RBOHs), cell differentiation, mitogenic functions and cell proliferation and differentiation (NOX4, NOXB) [[10], [11], [12], [13], [14], [15], [16], [17], [18], [19], [20], [21], [22]]. Mutations affecting function of the human NOXs cause severe diseases such as chronic granulomatous disease (NOX2), and NOX dysregulation has been attributed to cardiovascular, neurological disorders (NOX2, NOX4), and pulmonary diseases and cancers (NOX1 and NOX4) [[23], [24], [25], [26], [27], [28], [29], [30], [31], [32], [33], [34]].

**Fig. 1:**
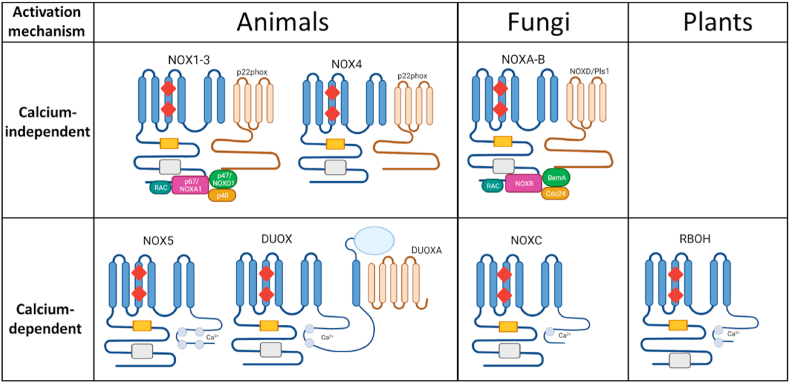
**Subunit composition of NADPH oxidases.** Activation mechanisms are classified here as ‘Calcium-independent’, *i.e.*: no direct binding of calcium is required for activation, and ‘Calcium-dependent’, *i.e.:* direct binding of calcium to the EF-hand domains is required for activation. The NADPH, FAD, and heme binding sites are outlined by the light grey, yellow and red shapes, respectively. (For interpretation of the references to colour in this figure legend, the reader is referred to the Web version of this article.)

NOXs can be categorized based on their dependence for activation by the direct binding of calcium or not. Calcium-binding NOXs include NOX5, DUOXs, NOXC and RBOHs. They are regulated through N-terminal calcium-binding EF-hand domains. They can possess either one (NOXCs), two (DUOXs, RBOHs) or four (NOX5s) calcium-binding sites (Fig. 1) [13,[35], [36], [37]]. Previous work showed that the C-terminal part of the regulatory domain is disordered in the resting state of human NOX5 [38]. When Ca^2+^ ions bind the apoenzyme, the disordered region undergoes a conformational change, resulting in an ordered scaffold that can interact with the dehydrogenase domain and stimulate activity. Calcium-binding NOXs typically do not require any accessory proteins for function. The only exceptions are DUOXs that need the transmembrane partner DUOXA - a maturation factor - to exert the enzymatic activity.

Calcium-independent NOXs generally interact with small transmembrane partners and are activated by cytosolic proteins which do not rely on the direct binding of calcium. Mammalian NOX1-4 are the most well-known and characterized NOXs of this group. They all share the same transmembrane partner, known as p22_phox_. NOXs 1-3 are activated by cytosolic proteins even though NOX3 shows some basal constitutive activity [39]. p47_phox_, p67_phox_, p40_phox_ and Rac1/2 are involved in NOX2 activation, whilst NOXO1, NOXA1 and Rac1 are found to activate NOX1 and NOX3 (Fig. 1) [[40], [41], [42], [43], [44]]. Intriguingly, NOX4, only requires p22_phox_ and the NOX4-p22_phox_ heterodimer is constitutively active [45]. In fungi, calcium independent NOXs, NOXA and NOXB, interact with membrane partners, called NOXD and Pls1, respectively [46]. In line with their metazoan counterparts, they also require additional cytosolic modulators to trigger enzymatic activity: BemA, NOXR, Cdc24 and Rac (Fig. 1) [13,14,46].

To gain insight into the emergence of differing activation mechanisms and their evolutionary relationships, we performed an in-depth phylogenetic analysis on the NADPH oxidase family in eukaryotes. Our results shed light on the key and complex evolutionary transitions that distinguish the several members of the NOX family. Furthermore, we demonstrate that the direct activation by calcium binding seems to be ancient in Eukarya and that the earliest eukaryotic organisms likely possessed several NOXs.

## 2 Materials and methods

1. Sequence collection

**Fig. 2:**
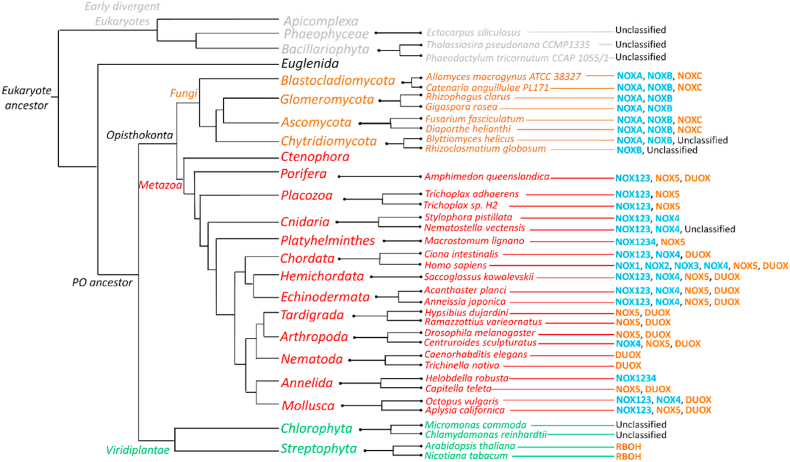
**Distribution of NADPH oxidases in Eukaryota.** For each kingdom, the figure outlines the fully sequenced representative genomes used to characterize the presence of the NOX subtypes. The calcium-dependent NOXs (orange), calcium-independent NOXs (turquoise) and unclassified NOXs (black) are shown. Early divergent eukaryotes, euglenida, viridiplantae, fungi and metazoan kingdoms are depicted in grey, black, green, orange and red, respectively. Opisthokonta represent the ancestor of fungi and metazoan kingdoms whilst the PO (Plant-Opisthokonta) depicts the ancestor of metazoan, fungi and plants, collectively. (For interpretation of the references to colour in this figure legend, the reader is referred to the Web version of this article.)

NOX sequences were first collected from different *phyla* of eukaryotic species to have a good representation of species with respect to the timeline taken in analysis (Fig. 2). Human NOXs were used as templates for protein BLAST searches. Two organisms per *phylum* that had a fully sequenced genome where vetted. This strategy ensures that we have detected all possible homologs in the chosen group of organisms. Also, some sequences located in isolated clades (for example, NOX1234 sequences from *Helobdella robusta* and *Macrostomum lignano*) were used as queries in homology searches. Of the total sequences collected, 16 sequences were from *Viridiplantae* (14 *Streptophyta* and two *Chlorophyta*), 87 from metazoans (12 *Tardigrada*, 7 *Arthropoda*, three *Nematoda*, five *Anellida*, seven *Mollusca*, 13 *Cnidaria*, two *Placozoa*, three *Porifera*, 11 *Echinodermata*, six *Hemichordata*, 16 *Chordata* and two *Platyhelminthes*), 35 from *Fungi* (11 *Blastocladiomycota*, seven *Glomeromycota*, six *Ascomycota*, one *Basidiomycota* and 10 *Chytridiomycota*) and six sequences from early divergent *Eukarya* (one *Phaeophyceae* and five *Bacillaryophyta*).

2. Dataset analysis and multiple sequence alignment

Once the dataset had been collected, a multiple sequence alignment (MSA) was performed using MAFFT v.7 [47]. The quality of the dataset and MSA was assessed by generating a Neighbour Joining (NJ) guide tree, using MEGA-X [48]. Some sequences not belonging to the canonical NOX clades were removed from the dataset, based on a sequence identity threshold <20% with at least one of the canonical NOX sequences. The Sequence Identity and Similarity tool (http://imed.med.ucm.es) was used to calculate pairwise sequence identity and similarity from the raw MSA. After several iterations, the working dataset was refined (132 sequences) and a MSA was constructed and trimmed (482 sites) (phylogenetic analysis was repeated adding seven cyanobacterial NOX5-like sequences; MSA with 139 sequences, 497 sites).

3. Phylogenetic inference

With the definitive MSA available, a substitution model was calculated with ProtTest v3.4 following the Akaike information criterion. The best-fit substitution model for our dataset was LG with a gamma distribution parameter equal to 1.347. A sequence subset was created, narrowing down to the *Opisthokonta* organisms (*Metazoa* and *Fungi*). Before carrying on with the generation of a tree, a reinforcement of this dataset was performed to especially amplify the population of unclassified sequences. After obtaining the MSA (112 sequences, 503 sites) and performing all the steps as already described above, the ProtTest analysis determined LG to be the best-fit substitution model with a gamma distribution value of 1.294. Phylogenies were inferred by exploiting the Maximum Likelihood (ML) method in PhyML v3.0 or RAxML v8.2.10 (500 bootstraps). Midpoint rooting was performed. Alternatively, the unclassified sequences were used as outgroup due to their sequence resemblance to ferric reductase enzymes. Both rooting strategies rendered the same tree topology.

4. Ancestral sequence reconstruction

The raw MSA generated for Eukarya phylogenetic analysis was re-trimmed more conservatively, to preserve all the specific domains and features of each NOX clade (132 seqs, 1783 sites). Ancestral sequence reconstruction was performed through the Maximum-Likelihood inference method in PAMLX v.4.9 [49,50]. Sequences were analyzed using an empirical amino acid substitution model (model = 3), four gamma categories and LG substitution matrix. The posterior probability distribution of ancestral states at each site was analyzed at nodes corresponding to all canonical NOX ancestors. The ancestor of all NOXs (node 151), the ancestor of all NOXs but NOX5 (node 152), the ancestor of all NOXs but NOX5 and NOXC (node153), the ancestor of RBOHs and calcium-independent NOXs (node 154), the ancestor of all calcium independent NOXs (node 155), ancestor of fungal calcium-independent NOXs (node 156), the ancestor of metazoan calcium-independent NOXs (node 176), the ancestor of NOX123 and the two NOX1234 sequences (node 185) and the ancestors of all canonical NOX clades (NOX5, node 243; NOXC, node 239; DUOXs, node 216; RBOHs, node 205; NOXA, node 157; NOXB, node 163; NOX123, node 186; NOX4, node 177) were targeted (Table S1). During the analysis of these ancestral sequences, additional MSAs were generated comparing the ancestral sequence of interest with its respective descendent sequences and including those predating the ancestor emergence. The lengths of the inferred ancestors were defined by the Fitch parsimony algorithm. The ancestral sequence inferred using the fully trimmed MSA (node 154) was used to generate an AlphaFold model representative of the catalytic core of the dataset.

5. Structural predictions

Analyses on some extant and ancestral sequences were performed using the PROSITE database of Expasy [51] for domains prediction (EF-hand domains, peroxidase-like domain, FAD-binding domain), the Metal Ion-Binding site prediction and docking server [52,53] to predict calcium-coordinating residues and AlphaFold2 Colab and Alphafold2 from COSMIC^2^ [54] to predict protein structure. AlphaFold and PDB-deposited structures were analyzed exploiting Chimera X [55,56]. Logos of the MSAs were generated in WebLogo [57,58].

## 3 Results and Discussion

1. Distribution of the NOX family in the Eukarya domain

Before attempting to build a phylogenetic tree of the NOX family, we first sought out to map their distribution within eukaryotes. The first eukaryotic organisms are estimated to have emerged approximately 2.1 billion years ago (bya) [59]. During Paleo- and Meso-Proterozoic periods (ranging from 2.5 to 1.0 bya), these organisms underwent several key divergences including the formation of the ‘early-divergent eukaryotes’ consisting of *phyla* such as the *Apicomplexa, Phaenophyceoe* and *Bacillariophyta*. Our analysis extracted unclassified NOX-like sequences from these genomes. Interestingly, these NOXs do not possess any EF-hand domains and likely do not require the direct binding of calcium for activation. They may potentially employ an entirely novel mode of activation. Similarly, also the *Euglenida phylum* does not contain any sequence clearly identified as a NOX (Fig. 2).

NOX enzymes are widely distributed among eukaryotes. The *viridiplantae* kingdom comprises the least NOX diversity of all eukaryotes. The *Streptophyta* species (land plants) are found to possess only RBOHs whereas the *Chlorophyta* (green algae) only possess unclassified NOX-like sequences (Fig. 2). Of note, despite the minimal NOX diversity in the *Streptophyta* organisms, RBOHs have been shown to accommodate a range of different biological processes [[15], [16], [17], [18], [19], [20]]. Fungi display greater NOX diversity. They typically comprise three different NOX members (except for *Glomeromycota* that possess only two) including NOXA, NOXB, NOXC and an unclassified NOX found in the *Chytridiomycota* (Fig. 2). NOXA and NOXB are present in all fungal *phyla* whilst NOXC is not strictly conserved. Finally, the metazoan kingdom comprises the widest repertoire of enzymes (Fig. 2). Both calcium-dependent and calcium-independent NOXs are present, except for the *Tardigrada* (only NOX5 and DUOXs) and the *Nematoda* (only DUOXs). Specifically, the chordates (back-boned organisms) possess all five NOXs and two DUOXs. Yet, not all metazoans contain NOXs since the *Ctenophora* do not exhibit any NOX member. However, missing sequences due to incomplete database record cannot be ruled out. Previous analyses already identified a similar subgroup distribution [4,60].

**Fig. 3:**
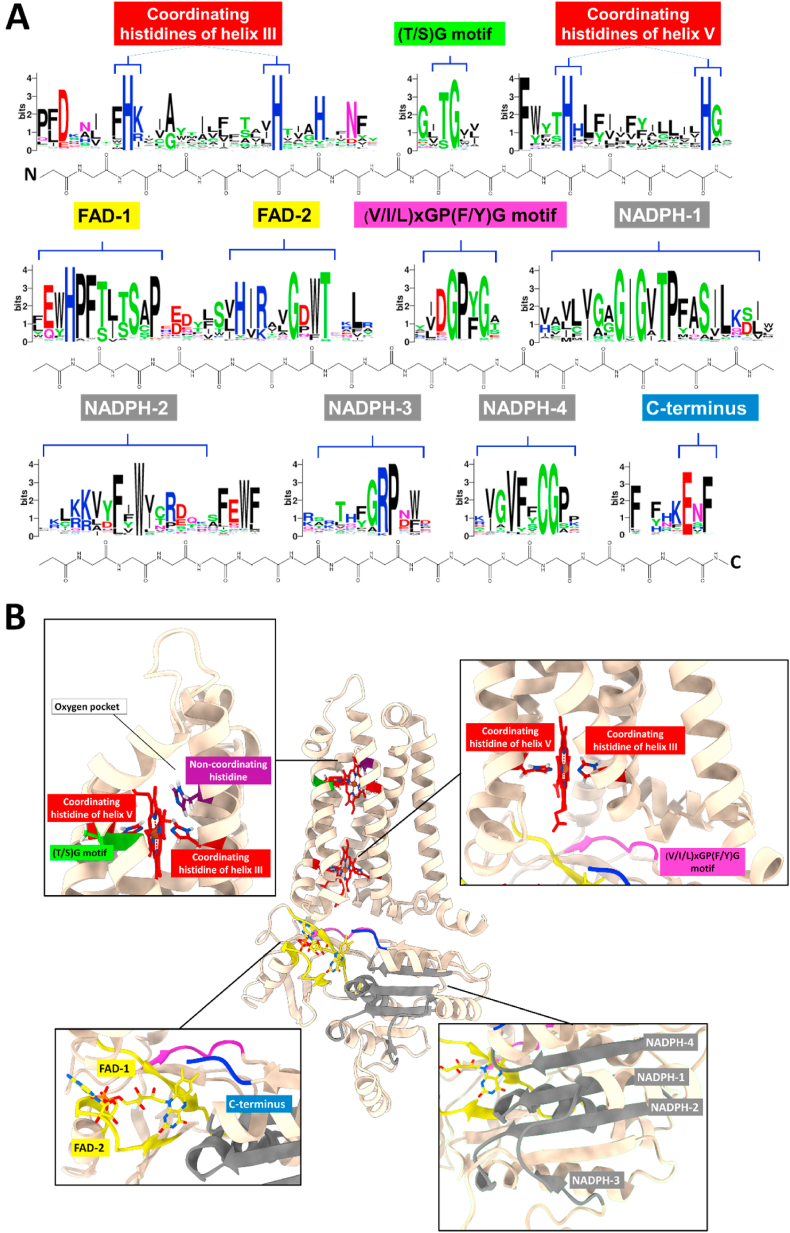
**NADPH oxidase catalytic core.(A)** Logo of the multiple sequence alignment describing the conserved motifs of the NOX sequences. **(B)** AlphaFold structural model of the catalytic core of the ancestral of RBOHs and calcium-independent NOXs, inferred through ancestral sequence reconstruction (see section 3, Results and Discussion). For a general description of the NOX architecture see [71].

All protein sequences included in our analyses presented at least 20% sequence identity with one of the canonical NOX sequences (NOX1-5, NOXA/B/C, DUOX, RBOH) and displayed the typical NOX features (Fig. 2, Fig. 3): six transmembrane helices for membrane integration, four iron-coordinating histidines for incorporation of two hemes, and a (V/I/L)xGP(F/Y)G motif that forms a β-strand located in the dehydrogenase domain at the interface with the transmembrane and the FAD- and NADPH- binding domains. Interestingly, all the canonical NOXs (and some unclassified sequences) were found to possess an extra conserved histidine in transmembrane helix III that does not coordinate the hemes (H119 in human NOX2 sequence). As it can be inferred from the NOX5 three-dimensional structures [61], this residue is part of the ROS-generating site on the outer heme of the protein. Furthermore, most of the sequences exhibit a well conserved (T/S)G motif (T178 and G179 in human NOX2 sequence), where the threonine/serine seems to have a stabilizing effect. The presence of the non-coordinating histidine in helix III and T/S residue in the (T/S)G motif have been previously observed to differentiate NOXs from the related ferric reductases (Fig. 3) [4].

2. Evolution of the NOX family

**Fig. 4:**
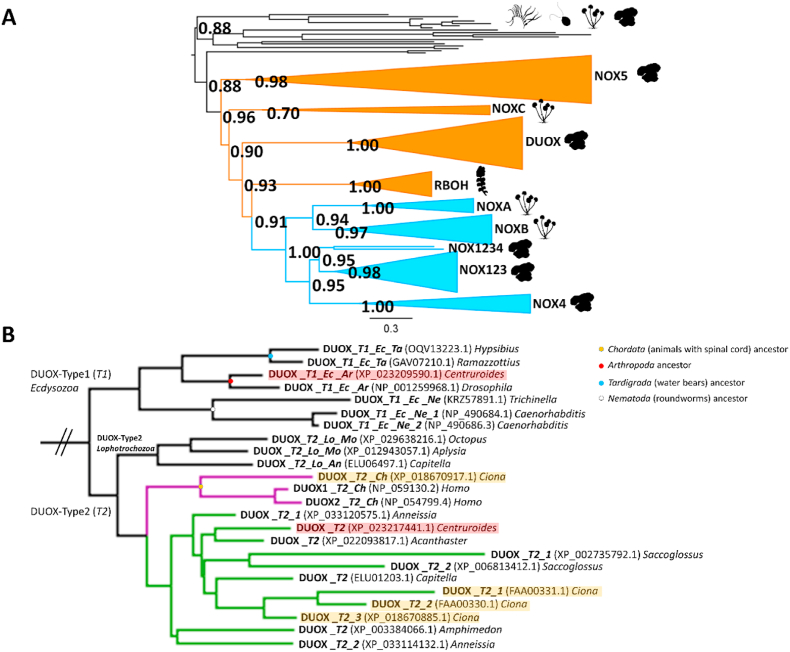
**Phylogenetic analysis of NADPH oxidase family. (A)** Phylogenetic analysis of eukaryotic NOXs by ML inference method. Orange clades correspond to calcium-dependent NOXs and cyan clades represent calcium-independent NOXs. Each clade is labeled with their respective NOX and the organisms (shown as silhouettes) that possess them. Branch supports are shown at key diverging points as transfer bootstrap expectations (TBE). Silhouettes represents:  early divergent eukaryotes,  Chlorophyta,  Streptophyta,  Fungi and  Metazoa. **(B)** Eukarya phylogenetic tree section focused on DUOX. The DUOX clade is divided in Type 1 and Type 2 subclades. *Centruroides sculpuratus* and *Ciona intestinalis* sequences are highlighted in red and yellow, respectively. (For interpretation of the references to colour in this figure legend, the reader is referred to the Web version of this article.)

The NOX family consists of eight discrete sub-families and varies in the modes of activation. To investigate their evolutionary history and dive into the emergence of the diverse modes of activation, we conducted a phylogenetic analysis within the Eukarya domain as shown in Fig. 4A. Fig. S1 extends the analysis to the cyanobacterial NOX enzymes confirming their monophyletic origin with metazoan NOX5 [4,9]. All classified NOX members partitioned into separate and distinct clades. Metazoan sequences formed four distinct groups with high support: NOX123 (Transfer Bootstrap Expectation, TBE = 0.96), NOX4 (TBE = 1.00), NOX5 (TBE = 0.99) and DUOX (TBE = 1.00). Fungal sequences grouped with high support values in three clades: NOXA (TBE = 1.00), NOXB (TBE = 0.96) and NOXC (TBE = 0.70), whilst *Streptophyta* sequences formed a single clade; RBOHs (TBE = 1.00). All unclassified sequences found in *Chlorophyta*, *Cnidaria, Chytridomycota* and early-divergent eukaryotes did not belong to any known/determined NOX subclass and likely diverged before all other NOX clades. By employing two alternative rooting strategies (see methods), the tree topology recovered was the same, indicating that the unclassified sequences predate the emergence of the eight NOX classes.

### 3.1 Evolutionary analysis reveals a calcium-dependent origin

The NOX phylogeny in Eukarya delineates a concrete separation between the NOXs regulated by the direct binding of calcium and those controlled by other factors, such as cytosolic proteins (Fig. 4A). The calcium-dependent NOXs emerged earlier during the evolution, whilst calcium independence is more recent. These events are supported by high TBE values: the first NOX system to diverge is NOX5 (TBE = 0.96), followed by NOXC (TBE = 0.90), DUOX (TBE = 0.93) and then RBOH (TBE = 0.91), respectively. During evolution, calcium-dependent NOXs diversified through a series of duplications, and thus many species are found to possess several paralogs (Fig. 2, Fig. 4A). They form a paraphyletic group, and its distribution does not follow the species tree.

DUOXs are divided in two main subclades, here denoted as Type 1 and Type 2 (Fig. 4B). These subclades are likely the result of a gene duplication event followed by losses in some specific phyla. The Type 1 DUOXs comprise only sequences from *Ecdysozoa* (common ancestor of *Arthropoda*, *Tardigrada* and *Nematoda*). The Type 2 subclade is more populated and shows a patchy taxonomic distribution. Human DUOX1 and DUOX2 are the result of a recent duplication. *Ciona* possesses four DUOX sequences: one clustering with the human DUOXs whilst the others group with DUOXs from *Anellida*, *Hemichordata*, *Arthropoda*, *Echinodermata* and *Porifera*. In general, many of the species analyzed tend to possess two or multiple DUOXs.

The greatest number of sequences found for a given species is held by the land plants. For instance, *Arabidopsis* and *Nicotiana* each possess six RBOH paralogs. Furthermore, the phylogenetic analysis portrays RBOHs as the youngest calcium-dependent system and the closest NOX relative to calcium-independent systems. This is commensurate with RBOHs having, on average, significantly higher sequence identities with the calcium-independent NOXs (>35%) as compared with NOX5 (>30%) and with the other calcium-dependent NOXs (<30%; Supplementary Information). As opposed to DUOXs and RBOHs, most of the metazoan species owning NOX5 possess only one sequence. An exception is the *Tardigrada phylum*, whose studied species contain a series of duplications forming a separated subclade (Fig. S2).

### 3.2 Analysis of the EF-hand domain in calcium-dependent NOXs

**Fig. 5:**
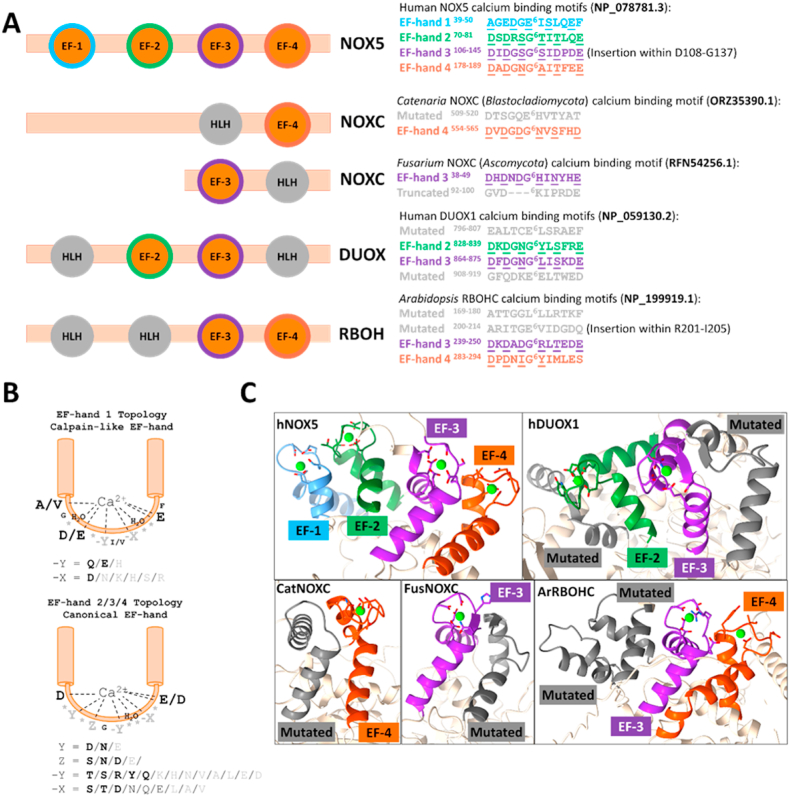
**EF-hand binding domain for calcium-dependent NOXs**. **(A)** On the left, schematic representation of the EF-hand binding motifs. HLH stands for helix-loop-helix. On the right, sequences of the EF-hand loops of representative sequences, including their positions in the sequence, for each NOX subtype. **(B)** Topology of EF-hand motifs present in NOXs. On the top, the calpain-like EF-hand found in NOX5. On the bottom, the canonical EF hands 2,3 and 4. The amino acid residues in bold are the most frequent residues found in the multiple sequence alignment. **(C)** AlphaFold models of the calcium-binding domains of human NOX5 and DUOX1, *Arabidopsis thaliana* RBOHC, *Catenaria anguillulae* NOXC and *Fusarium fasciculatum* NOXC. The AlphaFold model of the human DUOX1 is very similar to the experimentally determined structure [72] which lacks the electron density of one of the helix-loop-helix motifs.

The calcium-dependent NOXs exhibit varying profiles with up to four EF-hands in total, namely, EF-1, EF-2, EF-3, and EF-4 that span from the N-terminal to C-terminal, respectively. Using the phylogeny as a guide and by aligning the EF-hand domains, we sought out to determine which EF-hands were present for each NOX (Fig. 5A). NOX5 represents the most studied calcium-binding NOX and possesses all four EF-hands [35]. Exceptions included some sequences from *Platyhelminthes* and *Tardigrada* that lost the first two binding motifs and present a mutated EF-4 sequence that likely does not coordinate calcium. As previously observed by Kawahara *et al.* [60], aligning the EF-hand domains of all collected calcium-dependent NOX sequences against NOX5 illustrated that the two DUOX EF-hands align with EF-hands 2 and 3 whilst the RBOH EF-hands align with NOX5 motifs 3 and 4. The *Ascomycota* NOXC sequences just possess EF-hand motif 3 whereas the NOXC sequences from the *Blastocladiomycota* phylum contain only EF-hand motif 4.

The EF-hands feature a typical helix-loop-helix structure. The loop contains the calcium-coordinating residues. In NOXs, EF-hands 2, 3 and 4 are considered to be canonical EF-hands, whilst EF-hand 1 (only present in NOX5) corresponds to a non-canonical EF-hand motif and defined as calpain-like [62]. The canonical EF-hand coordinates the cation with residues 1, 3, 5, 7, 9 and 12 of the loop, conventionally defined as X, Y, Z, -Y, -X and -Z, respectively (Fig. 5B). X is generally an aspartate, while -Z always corresponds to an aspartate or glutamate and acts as a bidentate ligand, exploiting both oxygen atoms of the carbonyl side chain. The eighth coordination is performed by a molecule of water. The presence of the conserved glycine in position 6 is predicted to promote the loops flexibility [63]. Most of the NOX sequences analyzed satisfied these criteria for EF-hands 2, 3 and 4 (Fig. 5B). The only exception was the EF-hand 4 of RBOHs that misses key-coordinating residues on the second part of the loop; likely, this EF-hand coordinates calcium with less affinity. In the so-called calpain-like EF-hands (only present in the EF1 of the NOX5 sequences), calcium is coordinated instead by residues 1, 4, 6, 8 and 11, conventionally called X, Z, -Y, -X and -Z, respectively [62]. In the NOX5 sequences collected, X is either an alanine or a valine that coordinates calcium through the oxygen atom of the peptide carbonyl group; Z is a negatively charged residue, whilst -Z corresponds to a glutamate and coordinates in a bidentate mode. The coordination of residue Y, that is not present in calpain-like EF-hands, is substituted by a second molecule of water (Fig. 5B). Of note, the altered motifs of DUOX, NOXC and RBOHs are predicted by the AlphaFold models to maintain the helix-loop-helix secondary structure (Fig. 5C).

### 3.3 Calcium-independent NOXs share a monophyletic origin

With calcium-dependent NOXs forming a paraphyletic group, we inspected their metal independent counterparts in the phylogeny. They share a single common ancestor that diverged from the calcium-dependent group (TBE = 1.00) (Fig. 4A). In line with the species tree, two sister clades are observed, the one of fungal NOXs, NOXA and NOXB (TBE = 0.92), and the one of metazoan NOXs 1, 2, 3 and 4 (TBE = 0.94). Specifically, fungal NOXA and NOXB clades are conserved in all fungal phyla. All the species analyzed possess one sequence belonging to the NOXA group and one or more sequences grouping in the NOXB clade (apart from *Rhizoclosmatium* species that only have NOXB; Fig. S3). The metazoan calcium-independent NOXs are not as strictly conserved among all phyla. Two clades are identified within this group, one formed by NOX1, 2 and 3 sequences and the other exclusively containing NOX4 enzymes.

**Fig. 6:**
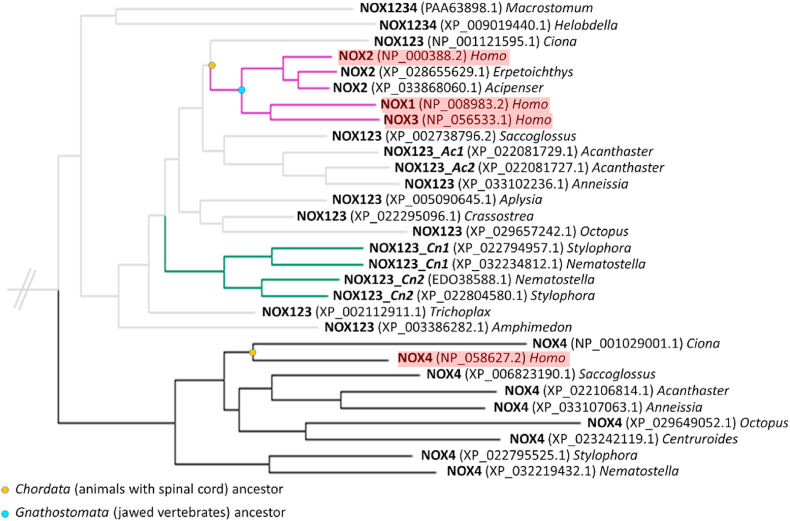
**Phylogenetic tree section of metazoan calcium-independent sequences.***Homo sapiens* sequences are highlighted in red. *Erpetoichthys calabaricus* (reedfish) NOX2 (XP_028655629.1), *Acipenser ruthenus* (sterlet fish) NOX2 (XP_033868060.1) and *Crassostrea virginica* (eastern oyster) NOX123 (XP_022295096.1) sequences were added to enrich the original dataset. They were selected since they resulted as BLAST outputs, using NOX1234 sequences (top sequences in the image) as BLAST queries. (For interpretation of the references to colour in this figure legend, the reader is referred to the Web version of this article.)

Phylogeny indicates NOX4 as the oldest classified metazoan calcium-independent system. NOX4 diverged as a separate clade from the NOX123 ancestor in the early stages of metazoan evolution and did not undergo any duplication events (Fig. 6, black clade). The NOX123 subtype is more broadly distributed than NOX4 that is only present in species that also possess a NOX123 sequence. The only exception to this pattern is the *Centruroides* species (*Arthropoda phylum*) that maintained NOX4 but lost NOX123 (Fig. 6). Furthermore, as originally shown by Sumimoto, Kawahara and co-workers, NOX1, 2, and 3, diverged inside the *Chordata phylum* [60,64]. Consistently, human NOXs 1-3 sequences all share a common origin corresponding to the vertebrate ancestor (Fig. 6, purple branches). Moreover, NOX1 and NOX3 are the most recent NOX subtypes developed in vertebrates [64]. Similarly to chordates, other metazoan phyla contain duplications of NOX123 sequences. For example, in *Cnidaria*, the two selected species, from *Nematostella* and *Stylophora genera,* encode for two NOX123 sequences (NOX123*_Cn1* and NOX123_*Cn2*, Fig. 6, green branches).

**Fig. 7:**
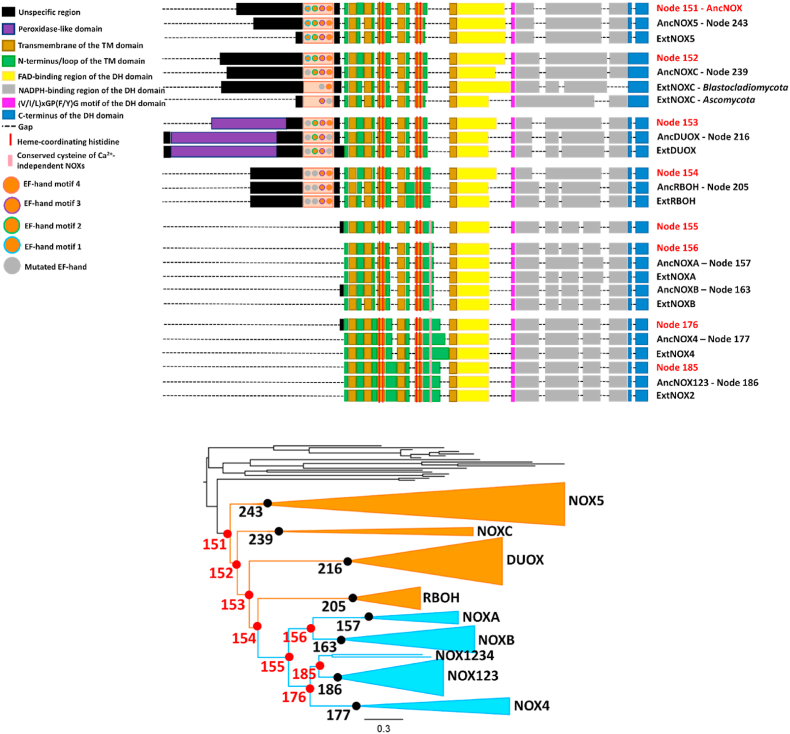
Domain architecture of NOX/DUOX ancestral (Anc) and extant (Ext) sequences.

Structurally speaking, calcium-independent NOXs are represented just by their catalytic core and they need protein partners to exert enzymatic activity. The absence of extra domains within the polypeptide chain makes it arduous to find a sequence fingerprint. Moreover, many changes in the sequence and length of the core, especially loops, are clade-dependent, so they do not delineate a feature that can be considered as essential for activation or protein-protein interaction. However, a cysteine placed in the E-loop was found as part of an insertion exclusively present in calcium-independent NOXs and conserved across all the sequences (only exception is the NOX4 sequence from *Saccoglossus*, where the cysteine is mutated to a serine; Fig. 7). When this cysteine is mutated to either a serine, an arginine or a glycine in human NOX2 (C244), it causes the genetic chronic granulomatous disease [[65], [66], [67]]. Moreover, the mutation of this cysteine in human NOX1 and NOX4 completely abolished and partially reduced enzymatic activity, respectively [68]. These data suggest a functional role of this residue in calcium-independent enzymes. Particularly, metazoan calcium-independent NOXs also possess a second conserved cysteine in the E-loop (C257 in NOX2) that has been found to be involved in enzymatic activity and was suggested to form a disulfide bridge with the previously mentioned cysteine [68]. However, this second cysteine is completely absent in the fungal enzymes.

3. Ancestral sequence reconstruction and structural analysis

With the aim to provide a comprehensive model that explains the taxonomic distribution of NOXs and shed light on the evolution of the alternative modes of activation, ancestral sequence reconstruction was performed at the key nodes in the phylogeny (Fig. 7). Our analysis reveals that the first members of the NOX class in eukaryotes were calcium-dependent enzymes (nodes 151, 152 and 153). Interestingly these diverged into different clades by the acquisition of additional domains, as DUOXs (node 216), or by losing some of the EF-hand motifs, as NOXCs, DUOXs and RBOHs. Hence, the differing number of EF-hand motifs exhibited by each NOX-type is probably due to mutation and deletion events with loss of calcium-coordinating residues and/or domains, rather than different acquisition events (Fig. 7).

Calcium-independent NOXs emerged later at a single event characterized by the complete loss of the N-terminal EF-hand motifs. By analyzing the predicted structure of the common ancestor of this group (node 155, Fig. 7), it can be speculated that it was probably an inactive or partially constitutively active protein. Consequently, this EF-hand domain-free NOX then supposedly gained a protein partner and cytosolic activators, as suggested by the similarities between NOX1-3 and NOXA/B [13,14,[40], [41], [42], [43], [44],^46^]. NOX4 however, does not require these cytosolic modulators. Therefore, the divergence of the NOX4 clade from NOXs 1-3 may well highlight the loss of capability for the enzyme to interact with the cytosolic modulators, instead of a gained function of the NOX1-3 ancestor (Fig. S4). Finally, a structural prototype of the common NADPH oxidase core of all eukaryotic NOXs was constructed (node 154, Fig. 3B). This shows that all structural key elements (discussed in section 1) were already defined at this early stage of evolution.

## 4 Conclusions

NOX-like enzymes were present at the very early stages of the eukaryotic evolution. It remains to be seen if and how these most ancient enzymes were regulated. The presence of unclassified NOXs lacking a calcium-dependent regulatory domain in the early-diverging eukaryotes indicate that calcium regulation might have been absent in the NOX-like precursors. On the other hand, our work demonstrates that the first well-classified members of the NOX class in Eukaryotes were calcium dependent (Node 151 in Fig. 7). NOX5 was the first clade to diverge, followed by the separation of NOXC (Node 152 in Fig. 7), DUOXs (Node 153), and the ancestor of plant RBOH, fungal NOXA/B and metazoan NOX1234 (Node 154). There are two possible interpretations regarding the emergence of RBOHs and calcium-independent NOXs. The first implies that very early events of duplication and divergence produced the two ancestors of the calcium-independent NOX1234AB and calcium-regulated RBOH, respectively (nodes 155 and 205 in Fig. 7). The ancestor of plants and *Opisthokonta* thereby already possessed both of them and during the speciation event that produced the *Viridiplantae* and the *Opisthokonta*, the former kept the RBOH ancestor, whilst the latter kept the NOX1234AB ancestor. Alternatively, a more parsimonious hypothesis would imply that the plant and *Opisthokonta* common ancestor possessed a single NOX1234AB-RBOH (node 154 in Fig. 7) that underwent enzyme diversification during the speciation events. In *Opisthokonta*, the enzyme lost calcium-dependency giving rise to NOXA and NOXB in fungi and the various NOX1-4 forms in animals. Homology searches shows that the distribution of cytosolic/transmembrane partners of calcium binding-independent NOXs (p47_phox_, p67_phox_ and p22_phox_) is restricted to opisthokonts and only to those species that comprise NOXs that do not directly bind calcium (Fig. S4). This suggests that when the EF hand domains were lost, alternative regulatory mechanisms were developed by employing cytosolic protein partners. The presence of NOXs responding to the different activation mechanisms can be interpreted by the need of fungi and animals to have a more finely regulated ROS production by these enzymes in certain cellular pathways, such as host defense and cell proliferation [10,11,13]. This hypothesis is also supported by the evolution of DUOXs, acquiring the DUOXA membrane-partner interaction to exert the enzymatic activity in presence of calcium [69]. This raises the question on NOX4-p22_phox_ heterodimer and its putative constitutive activity. We can infer random mutations made NOX4 insensitive to cytosolic modulators and acquired constitutive, though low [70], activity in a metazoan ancestor.

Our analysis clearly indicates that NOX-like enzymes were present at the very early stages of eukaryotic evolution. The common NADPH oxidase core of this family was already precisely defined and likely a functional enzyme. Our integral analysis suggests that the first mode of activation for the family in eukaryotes was depending on the direct binding of calcium and later the more-sophisticated regulation by protein-protein interactions emerged to fine-tune the differing roles on NOXs in redox signaling.

## Author contributions

M.M., C.N., S.M. performed all the sequence and bioinformatics analyses. M.L.M. guided the analysis. All authors conceived the experiments and wrote the manuscript.

## Declaration of competing interest

The Authors declare no conflicts of interest.

## Data Availability

Data will be made available on request.
