# Supplementary material for: Evolutionary and structural analyses of the NADPH oxidase family in eukaryotes reveal an initial calcium dependency
Source: Redox Biol. 2022 Aug 12;56:102436. doi: 10.1016/j.redox.2022.102436 (PMC9421330; doi:10.1016/j.redox.2022.102436)
Supplement: Multimedia component 1 [file mmc1.pdf]

Supplementary Information

Evolutionary and Structural analyses of the NADPH Oxidase Family in Eukaryotes reveal an initial calcium dependency

Marta Massari[1], Callum R. Nicoll[1], Sara Marchese[1],
Andrea Mattevi[1,*] and Maria Laura Mascotti[2, 3,*]

[1]Department of Biology and Biotechnology Lazzaro Spallanzani, University of Pavia, via Ferrata 9, 27100 Pavia, Italy

[2]Molecular Enzymology, Groningen Biomolecular Sciences and Biotechnology Institute, University of Groningen, Nijenborgh 4, 9747 AG Groningen, The Netherlands

[3]IMIBIO-SL CONICET, Facultad de Química Bioquímica y Farmacia, Universidad Nacional de San Luis, Ejercito de los Andes 950, D5700HHW San Luis, Argentina

*Correspondence to Andrea Mattevi, andrea.mattevi@unipv.it and Maria Laura Mascotti, m.l.mascotti@rug.nl

Supplemental Files

- *Supplementary_Eukaryota sequence list*: text file listing all sequences used for the analysis in fasta format.
- *Supplementary_SIAS*: excel file listing the pairwise sequence identities from the raw multiple sequence alignment.
- *Supplementary Eukarya phylogenetic analysis*: Newick tree format file picturing the phylogenetic analysis of eukaryotic NOXs.

Supplementary Figures and Table

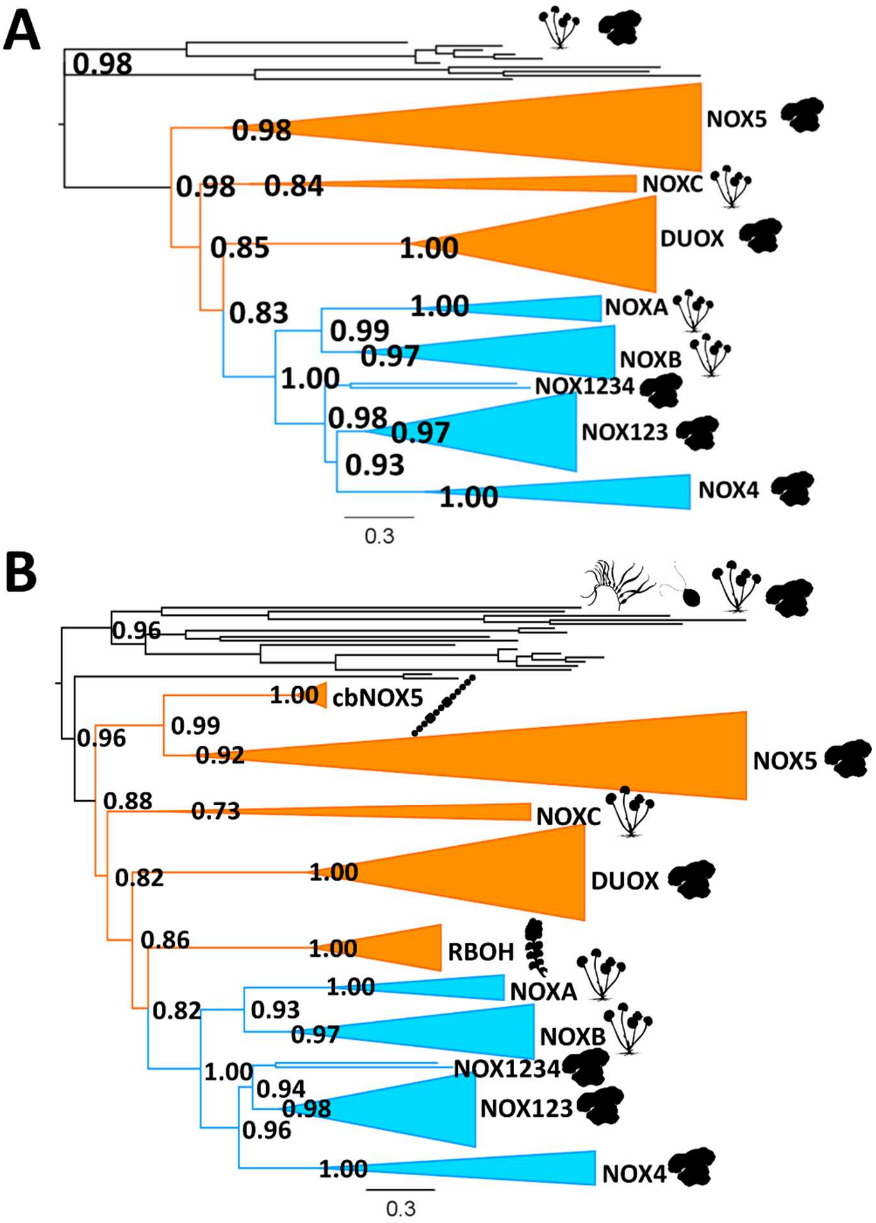

Figure S1. (A) Phylogenetic analysis of *Opisthokonta* NOXs by ML inference method**. (B)** Phylogenetic analysis of *Eukarya* NOXs and cyanobacterial NOX5-like sequences by ML inference method. The evolution of the NOX family is shown with orange clades corresponding to calcium-dependent NOXs and cyan clades representing calcium-independent NOXs. Each clade is labelled with their respective type NOX and the organisms (shown as silhouettes) that possess them. Branch supports are shown at key diverging points as transfer bootstrap expectations (TBE). Silhouettes represents: early divergent eukaryotes, Cyanobacteria, Chlorophyta, Streptophyta, Fungi and Metazoa.

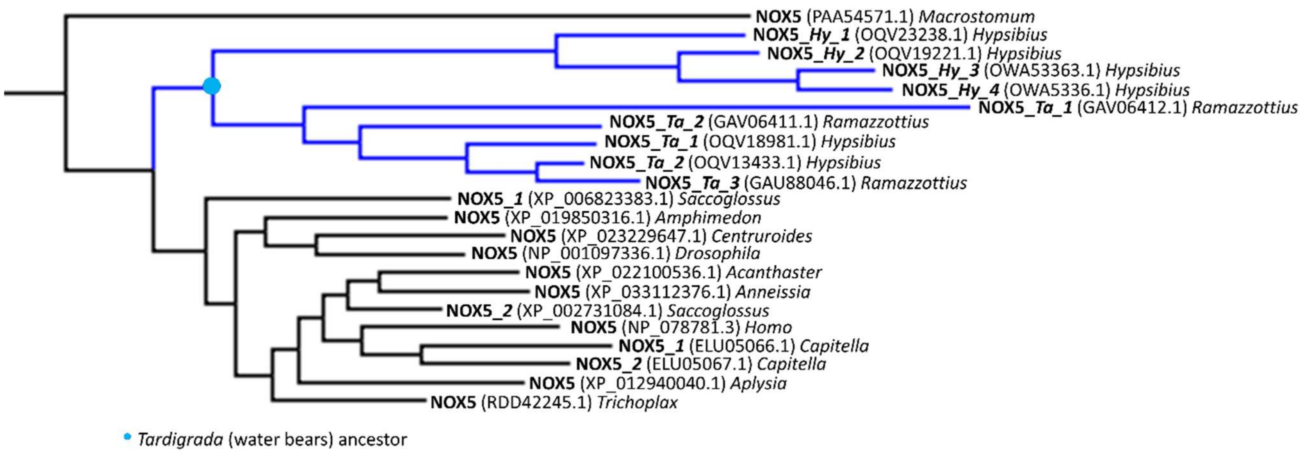

Figure S2. *Eukaryota* phylogenetic tree section focused on NOX5.

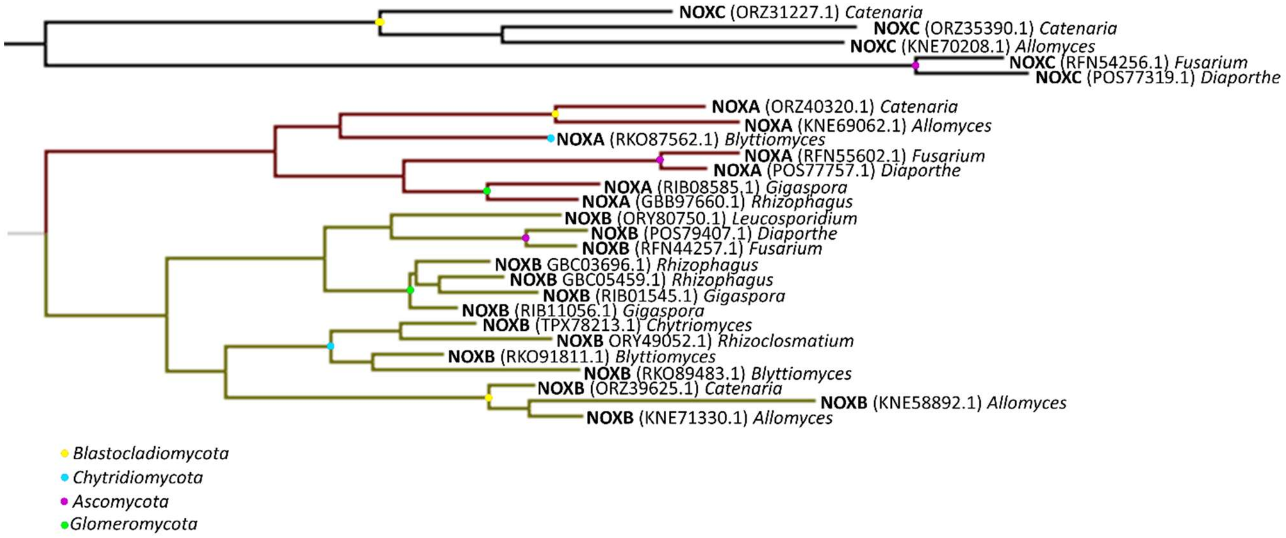

Figure S3. *Eukaryota* phylogenetic tree section focused on fungal NOXC (top; black clade) and fungal calcium-independent sequences (bottom; brown clade for NOXA and gold clade for NOXB). *Leucosporidium creatinivorum* NOXB (ORY80750.1, *Basidiomycota*) and *Chytriomyces confervae* NOXB (TPX78213.1) sequences were added to enrich the original dataset.

A

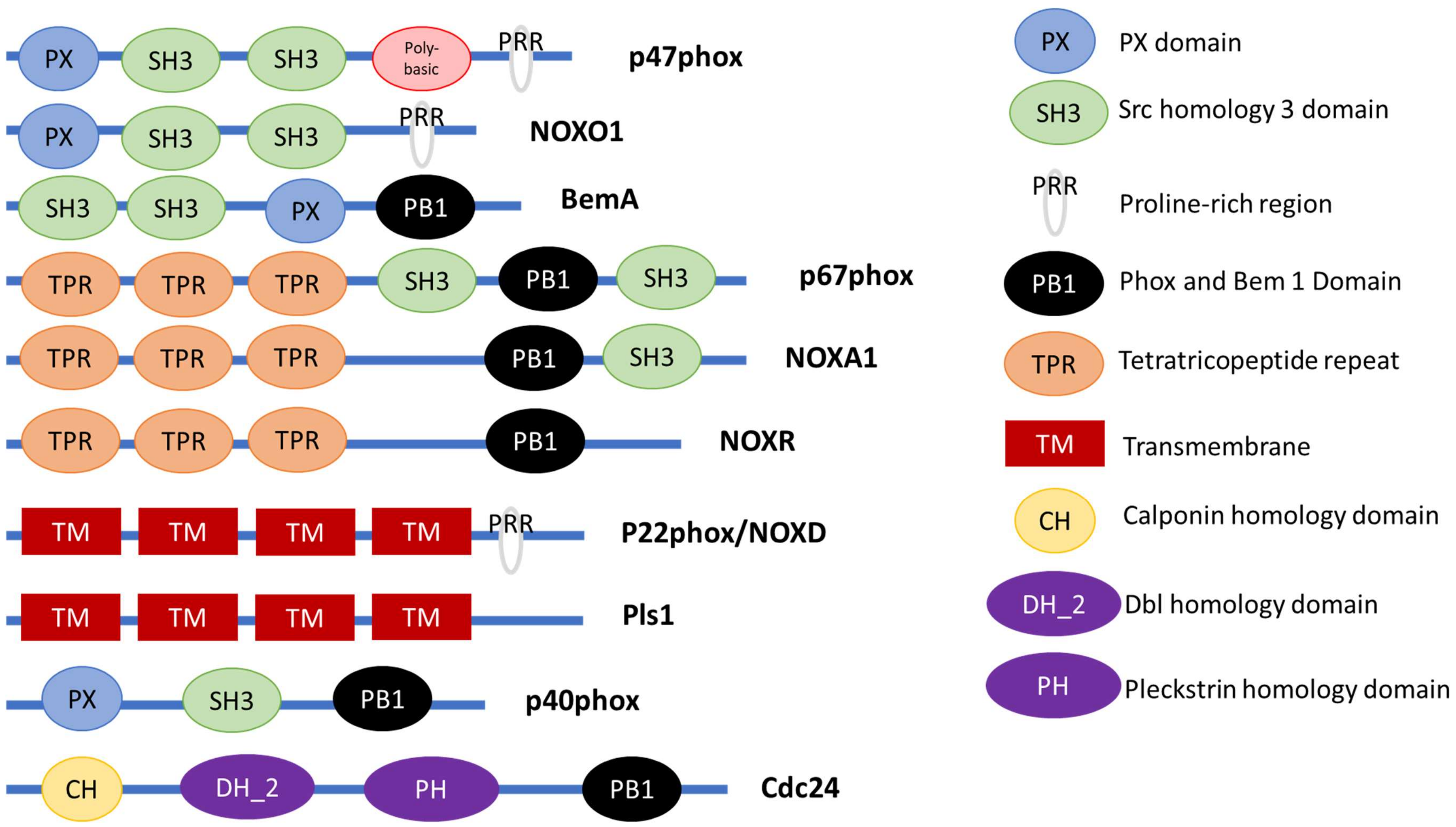

PX
SH3
SH3
Poly-basic
PRR
p47phox
PX
SH3
SH3
PRR
NOXO1
SH3
SH3
PX
PB1
BemA
TPR
TPR
TPR
SH3
PB1
SH3
p67phox
TPR
TPR
TPR
PB1
SH3
NOXA1
TPR
TPR
TPR
PB1
NOXR
TM
TM
TM
TM
PRR
P22phox/NOXD
TM
TM
TM
TM
Pls1
PX
SH3
PB1
p40phox
CH
DH_2
PH
PB1
Cdc24
PX
PX domain
SH3
Src homology 3 domain
PRR
Proline-rich region
PB1
Phox and Bem 1 Domain
TPR
Tetratricopeptide repeat
TM
Transmembrane
CH
Calponin homology domain
DH_2
Dbl homology domain
PH
Pleckstrin homology domain

Figure S4.

B

		ORGANIZERS			ACTIVATORS			TRANS-MEMEBRANE SUBUNIT			OTHER REGULATORS	
**Phylum**	**Species**	**NOXO1-like**	**p47phox-like**	**BemA-like**	**NOXA1-like**	**p67phox-like**	**NOXR-like**	**p22phox-like**	**NOXD-like**	**Pls1-like**	**p40phox-like**	**Cdc24-like**
**Early divergent eukaryotes**												
Phaeophyceae	*Ectocarpus siliculosus*											
Bacillariophyta	*Thalassiosira pseudonana CCMP1335*											
	Phaeodactylum tricornutum CCAP 1055/1											
Viridiplantae												
Streptophyta	*Arabidopsis thaliana*											
	Nicotiana tabacum											
Chlorophyta	*Micromonas commoda*											
	Chlamydomonas reinhardtii											
**Fungi**												
Blastocladiomycota	*Allomyces macrogynus ATCC 38327*			✓ (no PB1 domain)			✓ (+ SH3 domain)		✓			✓ (no CH and PH domains)
	Catenaria anguillulae PL171			✓ (no PB1 domain)			✓ (no TPR domain)					✓ (no CH, PH and PB1 domains)
Chytridiomycota	*Blyttiomyces helicus*			✓ (no PX and PB1 domains)	✓ (no SH3 and PB1 domains)		✓ (no PB1 domain)		✓			✓ (no CH and PB1 domains)

	Rhizoclosmatium globosum						✓ (no PB1 domain)		✓			✓ (no CH, PH and PB1 domains)
Ascomycota	*Fusarium fasciculatum*											
	Diaporthe helianthi			✓						✓		✓ (no CH and PH domains)
Glomeromycota	*Rhizophagus clarus*			✓ (no PB1 domain)			✓			✓		✓ (no CH domain)
	Gigaspora rosea			✓			✓			✓		✓ (no CH domain)
**Metazoa**												
Porifera	*Amphimedon queenslandica*		✓			✓ (no SH3 domain)		✓			✓ (no PB1 domain)	
Placozoa	*Trichoplax adhaerens*		✓ (no SH3 domain)				✓ (no PB1 domain)				✓ (no SH3 and PB1 domains)	
	Trichoplax sp. H2		✓				✓ (no PB1 domain)	✓			✓ (no SH3 and PB1 domains)	
Cnidaria	*Stylophora pistillata*		✓					✓			✓ (no PB1 domain)	
	Nematostella vectensis		✓			✓ (no SH3 and PB1 domains)		✓			✓ (no PB1 domain)	
Platyhelminthes	*Macrostomum lignano*		✓					✓			✓ (no PB1 domain)	

Chordata	*Ciona intestinalis*		✓			✓ (no SH3 and PB1 domains)		✓			✓ (no PB1 domain)	
	Homo sapiens	✓	✓		✓	✓		✓			✓	
Hemichordata	*Saccoglossus kowalevskii*		✓			✓ (no PB1 domain)		✓			✓	
Echinodermata	*Acanthaster planci*		✓			✓ (no PB1 domain)		✓			✓ (no PB1 domain)	
	Anneissia japonica		✓			✓ (no SH3 and PB1 domains)	✓ (no PB1 domain)	✓			✓ (no PB1 domain)	
Arthtopoda	*Drosophila melanogaster*											
	Centruroides sculpturatus											
Tardigrada	*Hypsibius dujardini*											
	Ramazzottius varieornatus											
Nematoda	*Caenorhabditis elegans*											
	Trichinella nativa											
Annelida	*Helobdella robusta*											
	Capitella teleta		✓								✓ (no PB1 domain)	
Mollusca	*Octopus vulgaris*											
	Aplysia californica		✓					✓			✓ (no PB1 domain)	

Figure S4. Domain organization (**A**) and distribution among eukaryotic species (**B**) of the proteins regulating the NOXs that do not directly bind calcium. All the species containing NOX1-4, NOXA-B are highlighted in light blue. *Capitella teleta* is exceptional in that it does not own any NOX1-3 or NOX4 but contains a p47/40phox-like sequence. The search was performed using the following reference sequences: human NOXO1 (NP_751908.1), human p47phox (NP_000256.4), *Gigaspora rosea* BemA (RIB26013.1), human NOXA1 (NP_001242996.1), human p67phox (NP_000424.2), *Gigaspora rosea* NOXR (RIB16941.1), human p22phox (NP_000092.2), *Allomyces macrogynus* NOXD (KNE56820.1), *Gigaspora rosea* Pls1 (RIB25271.1), human p40phox (NP_000622.2), *Gigaspora rosea* Cdc24 (RIB18608.1). The listed sequences have at least 30% query cover on BLASTp with one of the reference sequences. The presence of SH3, PB1, PX, TPR, CH, DH_2, PH domains was verified with the PROSITE tool from Expasy (https://www.expasy.org).

Table S1. Sequences and posterior probabilities (PP) of ancestral reconstructed proteins.

>Node 151
MAKALNNDWGSVGSPLTRKVPPSYADGVYKPAGAKRPNPRSASNDSRGGRLLPLLRTRHDKNTSLLFRLGDPRGNENPAIRALGLLFYRYHNYLAELVARQVFSKDRRWTIA
TLQKVILHEPSTSQLFQLAAMEEELILGMASQLTERDDNVIVEDLRGRETPGLLERFKLLHNNDASNLGLLERFEFLKGHGKGVELKEVGQKELLLSAETDEKWLKWIEKRF
KEVAAEDAQLDREEFAEALGMKPSSLFAERLFSLVDKDRSGSISFRELLDALNMLTHGSSDDKLKLLFDMYDVDGDGRIDREELKQVLRSCMEANGLSLSPEQLDELVNAIL
ESLDADGDGEISFEDFKVLLRKYPEELANLSLSAASWLKPPEKRRKSVPARARKTLLKAYAERKKSRTLTESLKPEKEKSRVRTPWRRLRRYLQNHGPKIVFLVLFLLINLG
LFVWSFYQYKNSPEYYSLRKIMGYSVAIARGCGSVLNFNCAFILLPMCRNLLTWLRSTRLNRYIPFDKSITFHKLIGWVIVFFSLVHTVCHLFNFSHLAEDPSADLESLLRR
LPNLTYYADALPPKFGYGLFAKSIAGLTGWILVVVLVIMFVTSLPFVRRSHYFEVFWYTHLLFIPFIVLLILHGSGALVKPPTFWYYLVVPAVLYVLERILRVKCLRLSRYR
KVTIVKVELLPSNVVELVMKRPPGFKYKPGQYVFINVPAISRFEWHPFTISSCPEQDDHLSLHIRAVGDWTKKLYELFDENEGEEKPLLGDPDDNLQEETARKLPTKPTLSR
KTRRPGKLGTLLRLVHVEVYIDGPYGAPSQDFFKYEHAVLIGAGIGVTPFASILKDLVHRHKSGRQSCPSANTDWSDDVPGSKMKLRKVDFFWVTRDQKSFEWFADLLNELE
EEQAEEGNLDDFLEIHIYLTSARGKSDLKSVMLHMALELAHKKNGRDLITGLRTRTHYGRPDWDKIFEKIAEEHNGSKVGVFYCGPPALAKTLRKACQKYNKKEGTRFEFHK
ENF
PP = 0.60

>Node 152
MAKAVPPSYADGVPNPRSASNGLPSLRNRTTLFVYFGEILPATGPGCPPDSRGGRLLPLLRTRHDKNTGLSPIYSTSEAWADALRSFKGGKLAADDGAEFPPENTIGLPLAN
PPPPATAKLRGNENPAIRASRPLGLLFYRYHNYLAERIARQHPDKDRRWTIATLQKIILHEWLPAFLGHLQPSTSTLVPPGTFLRNGACHFRGTLPALRLCRDDNVIVEDLR
GRGLPDYPPLTNWEDINPLLHNNDASNLDLYPGGLLETESDPPGPLEFLKGHGKGVELKEVGQKELLLSAETDEKWLKWIEKRFKEVAAEEELKDVLDAQLDREEFAEALGM
KPSSLFAERLFSLVDKDRSGSISFRELLDALNMLTHGSSDDKLKLLFDMYDTDGDGRIDREELKQVLRSAMEANGLSLSPEQLDELVVKPSAHHVLHTDHDNDAGNAILESL
DADGDGEISFEDFKVLLRKYPEELANLSLSAASWLKPPEKRRKSVPARARKTLLKAYAERKKSRTLTESLKPEKEKSRVRTPWRRLRRYLQNHGPKIIFLVLFLLINLGLFV
WSFYQYKNSPEYSLRKIMGYSVAIARGCAAVLNFNCAFILLPMCRNLLTWLRSTRLNRYIPFDKSITFHKLIGWVIVFFSLVHTVCHLCFNFSHLAEDPTADLESLLRRTYY
SDALPPKFGYLLFAKSIPGLTGWILVIVLVIMFVTSTPAVRRSHFEVFWYTHHLFIPFIVLLILHGSGALIKPPTFWYYLVVPAVLYVLERLLRVKCFRSRYRKVTIVKVEL
LPSNVVELVMKRPPGFKYKPGQYVFINVPAISRFEWHPFTITSCPSEDDYLSVHIRAVGDWTRKLYELFDENEGEEKPLLGDPDDNLQEALVEPPAGGKSGRETARKLPTKP
TALLRLVNVEVYIDGPYGAPSQDFFKYEHAVLIGAGIGVTPFASILKDLVHRHKSGRQSCPSANTDWSDDTSEEPPKKDESGDGAPGSKMKLRKVDFFWVTRDQKSFEWFAD
LLRELEEEQAEEGNLDDFLEIHIYLTSARGKSDLRSVMLHMALELHAHKKNGRDLITGLRTSRTHYGRPDWDKIFEKIVEEVLKLRAEEHPNGSKVGVFYCGPPALAKTLRK
ACQKYNKKERRGEGGTRFEFHKENF
PP = 0.59

>Node 153
MARAVPPSYADGVQPAGANRPNPRSVSNELFKGPDGLPSLRNRTTLFVFFGEILPATGPGCPDSRGGRLMPLLRTRYDRNTGLSPNNPDEQLNEVTLWIIYSTSEAWADALR
SFKGGRLAADDGAEFPPENTIGLPLANPPPPATAKLRGNENPALLAPLGLLFYRWHNYLAERIARQHPDWSDKDRRWTIATLQNIILHEWLPAFLGHLHPSTSTLVPPGTYL
RNGQCHFRGTLPALRLCRDDNVIVEDLRGHVFGPLSFSRRDLTLGLPDYPPLTNWEDINPLHNNDLNNIDLYPGGLLETESNGPGTIIRDQFLRLRDTEEEIEEIRSLTLLA
YLLARRRERKQKKLDRARKGAKRSLRKLKKESTEEFAALEWLGHKEASRPVEVKLGPEATIRVLNRKGELLRTIDLKSLEAVSLLLSANKDRPLLLLRIPKEYDLVLRFDNE
EERNKFIEELEEFLKGHGKGVELRELGQKELLLSAETKEKRLKWIERYFKEVAAEAFNLDEDEDSKKLENAEELKDVLDAQLSREEFAEALGMKPSSLFAERLFSLVDKDRN
GSISFRELLDALNLLTHGSTDDKLKLLFDMYDTDGDGRISREELKQVLRGLMEANGLSLSEEQLDELVNAILKSLDADGNGEISFEDFKVLLRKYPEELANLSLSAASLLKP
PEKRRKSVPARARKTLLKAYAERKKSRTLTESLKPKKERSRVRTPWRRLRRYLQNHGPRIFFLVLFLVINIGLFVWSFYQYKNSPEYYSLRKIMGYSVAIARGAAAVLNFNC
ALILLPMCRNLLTWLRSTYLNRYIPFDKHITFHKVIAWVIVFFTVVHTVCHLCFNFSHLAESPSSDLESLLRRVYYSDTLPPKFSYLLFAKSIPGLTGVILVIVLVIMFVTA
TPAVRRSGFEVFWYTHHLFIVFYVLLILHGSGALIKKPTFWYYLVVPAVLYVLERLLRVKRSRRKVTIVKVELLPSNVVELQMKRPPGFKYKPGQYVFINCPAISRFEWHPF
TITSAPSEDYLSVHIRAVGDWTRKLRELFDEDEGEEQPAAVEPPVGGKSGRETARKFPTIYIDGPYGAPSQDFFKYEVAVLVGAGIGVTPFASILKDLVHRHKSNRQSKMKL
RKVYFFWVTRDQKSFEWFADLLRELEEQQAEENLDDFLEIHIYLTSAYDKSDLRSVMLHMAQEHAHKKNGRDLITGLRSRTHYGRPDWDKIFEKIREEHPNGSKVGVFYCGP
PALAKTLRKACQKFNKKEGEDGTRFEFHKENF
PP = 0.57

>Node 154
MSPLTKPVGDKRPNPRSASNELFEGPDGLGSSGPGCPGGKLLPLLRTRYDRNTGLSPNNPDEQLNEVTLWLRDDNVIVEDLEGKVFGPLSFSRRDLTLLNIMRGINPDLYKE
NPELGLLETEANGPGPLFTSIIRDQFLRLRDTEEELEEIRSLTGLAYLLARRRERKQKKKLDRARKGAKRALRGLRLRFLLSAETKEKRLKWIEKYFKEVAAEDYLDAQLSR
EEFAEALGMKPSSLFAERLFSLLDKDRNIKTGSISFEELLDALELLTHGSTDDKLKLLFDMYDKNGDGRISREELKEVLLSAMEANGLSLSEEQLDELVNAILESLDADGSG
KISFEEFKVLLRKYPEELTNLSAYEERKKSRTLTESLKPKKERSPVRSPWRRLRRYLQNHGPRIFFLVLWLVINIGLFVWSFYQYKNSPEYYTLRKIMGYSVAIARGAAAVL
NFNCALILLPVCRNLLTWLRSTYLNRYIPFDKNITFHKVIAWTIVFFTVVHTVAHLCFNFSRLAEAPSSDLESLLVYYSDTQPPKFTYLLFVKTVPGLTGVIMVIVLVIMFT
TATPAVRRSHFEVFWYTHHLFIVFYILLILHGAGAFIKKQWGGPTTWMYLVVPAVLYVLERLLRVLSRYRKVTIVKVVLHPSNVIELQMKKPGFKYKPGQYVFINCPAISRF
EWHPFTITSAPEDDYLSVHIRAVGDWTEKLRELFDEDEGEEQELAVEPPVGGKSGRETARKFPEIYIDGPYGAPSQDFFKYEVAVLVGAGIGVTPFASILKDLLHRHKNDKS
PMKLRKVYFFWVTRDQNSFEWFADLLQELEEQQAEENLDDFLEIHIYLTSAYDESDARSVMLHMAQELAHTKNGRDLITGLRSRTHYGRPNWDKIFEKIAEKHPGTKVGVFY
CGPPALSKTLRKACQKFSKKGEDGTRFYFHKENF
PP = 0.61

>Node 155 – Ancestral Ca^{2+}-independent NOX
MAKRPSKAAKRVKSPWRRFRSWLVNEGPRLFFLIIWLVINIGLFVYTFYQYKNSPEYYTLRTLLGYSVAIARGAAAVLNFNCALILLPVCRNLLSWLRSTFLNRYIPFDKNI
TFHKVIAWTIVFFTLVHTVAHCFNFYRLAEAYSSDADSYSNTDPHLLFVKTVPGLTGVIMVIVLILMVTSATEAVRRSHFEVFWYTHHLFIVFFILLILHGAGCFIKSQTNP
VCRGPTTWMYLIAPAVLYVLERILRVFRSRRKVTIVKVVMHPSNVIEIQMKKPGFKYKPGQYVFINCPSISRFEWHPFTLTSAPEDDYLSVHIRVVGDWTEKLAKLLGADEG
ERETARKLPEIYIDGPYGAPSEDVFKYEVAILVGAGIGVTPFASILKSIWYRHNQDNSPMKLRKVYFFWVCRDTQSFEWFADLLQELEEQNLPDFLEIHIYLTGGWDENEAR
NIMLHETKNDRDAITGLRSRTHYGRPNWDKIFKKIAESHPGTNVGVFFCGPKALSKTLHKACNKYSKAEDGTRFYYHKENF
PP = 0.75

>Node 156 – Ancestral fungal Ca^{2+}-independent NOX
MANKFKNWMVNEGPRLFFFILWLVIHVGLFVYSFFNYKNSPELTTLRTLLGYSVAIARGAAAVLNFDCALILLPVCRNLISWLRSTFLNRFIPFDKNITFHKCIAWAIVFFT
LVHTVAHYFNFYKVAEALSGPATALQLHFVTWPGLTGHIMLIVLFLMVTSATEKVRRSHFEVFWYTHHLFIVFFLCLLFHGAGCFIKSQTNPLCRGGPTTWKYLIASGVLYL
LERILREKKGRRKTTITKVVMHPSKVIEIQMKKPSFKYKPGQYIFINCPEISRFQWHPFTLTSAPEEDYISVHIRVVGDWTKALAKLLGCDEGDEEELENPRKLPEIMIDGP
YGAPSEDVFKYEVAILVGAGIGVTPFASILKSIWYRHNNPNSPMKLRKVYFFWVCRDTESFEWFQSLLQTLEEQNLDDFLEIHIYLTGGLDEDEIKNIMLNDTGNDKDAITG
LRSRTHYGRPNWDKIFKKIRDSHPGTNVGVFFCGPKALSKTLHKACNKYSSAGTRFYYHKENF
PP = 0.77

>Node 157 – Ancestral NOXA
MAASLFRKEFAPLFFWIFWLAFHIGLFVYGFFKQRDDPELRTLNSLGFSVWISRGAGLVLAFDSALILLPVCRNIISWLRSTFLNRFIPFDENIFFHKCVAYSMLFFTLVHT
TAHYVNFFKVEEQLPAPAWQIHYTTWAGITGHIMLILMFLMYTSATHKIRQQSFETFWYTHHLFIVFFLCLFFHGVGCFVKTTENGRCKGYFSWRYTIWSGVIYLLERVLRE
KCIRQPTTITKVVMHPSKAIEIQFDKPSFKYKPGQYLFINVPEISSFQWHPFTITSCPEEPYVSVHIRLVGDWTKALAKVLGCDEGDGVDPLENPKELPKLRIDGPYGAPAE
DVFKYEVAILVGAGIGVTPFASILKSIWYRYNRKAPMKLRKVYFFWVCRDTESFEWFQSLLKTLEEQQAEAAPSGFLEIHIYLTGKLDEDEIQNIVLNDVGNDYDPLTDLRS
RTHYGRPNWDRIFSKIRDAVLSGRYLPGAEAHQKTNVGVFFCGPAALAKTLKKACNKASTEGVKFHFHKEHF
PP = 0.79

>Node 163 – Ancestral NOXB
MSNSYNLQKKTSQKIRLIKKPESLWEKFENWMVNEGPKRFFFVLWILLHVGLFAYSFYNYQTSPNLTTLRTVLGYSLAIARSAAAVLNLDCALILFPVCRNLISLLRTTFLN
RFIPFDKNITFHKCIAWAIVFFTLVHTVAHYYNFYKVALALNGGVVAWLQLNFVTGPGLTGHVMLIALFLMVTSATEKVRRAHFEIFWYTHHLFIVFFAALLFHGAFCFIKA
DKNPLCRGGPTFWKYWIASGVLYLVERILREKKGRRKTYISKVVMHPSKVVEIQIKKPSCTTRAGQYIFINCPEISPFQWHPFTLTSAPEEDYISVHIRVVGDWTKAFAKAL
GCNFGDEDEANKDRENPKSLPEIMIDGPYGSASEDVFKYEVAILVGAGIGVTPFASILKSIWYRMNHPNSKMKLRKVYFFWVCRDTDSFEWFQSLLMTIEEENLDDFLEIHT
YLTGGLKPDEIKNIMINDAGGGKDAITGLRSRTHFGRPNWDKIFKGIREAHPGTDVGVFFCGPKALSKTLHKACNKWTDAGEDGTRFYYGKENF
PP = 0.78

>Node 176 – Ancestral Metazoan Ca^{2+}-independent NOX
MAKRFRSWLVNEGPRYLVLIIWLVINVVLFVYTFYNYKTSPEYYYLRTMLGYSLAIARGSAAVLNFNCALILLPVCRNLLSFLRGSCQVCSRNVRRLLDKNITFHKVIAWMI
CFHTAIHVVAHCFNFERFAESYNSSADELNPVRYSNTDPHLLFVKTVPGLTGVIMTLVLILMVTSSTEAVRRSHFEVFWYTHHLFIIFFILLVVHGMGGVIKRQTNLDEHNP
PVCHNNKTRWGNAKECKPVPQFAPSGPTTWMWVVAPMVLYVLERLLRFRKHYRKVTIVKVVQHPSNVIEIQMKKKGFKAKPGQYVFINCPSISRFEWHPFTLTSAPEDDYFS
VHIRIVGDWTERLAKLLGADEGEEQERETARKFPEIYIDGPFGSPSEDVFKYEVAILIGAGIGVTPFASILKSIWYRHNQDNQGMKLKKVYFFWICRDTQSFEWFADLLQEL
EQQMQEQNMPDFLEIHIYLTGGWDENQARNIMLHEDDDKDAITGLRSRTHYGRPNWDKIFKKIAESHPGTNVGVFFCGPKALSKTLHKMCNKYSSAEDGTRFYYNKENF
PP = 0.78

>Node 177 – Ancestral NOX4
MATAGLKRTARLFRSWLVNEGLKYLLLIIWLGINVLLFWRTFYQYKNGPEYYYLHQMLGYSLCWSRGSAAVLNFNCALILLPMCRNLLSFLRGSHKVASRRVRRLLDKNITF
HKVCAVTICLAAAVHVVAHLVNAKRFSENYNSDYPELNVARYRNEDPLKLLTKTVPGLTGILMMLVLFLMITSSTKAVRRSNYEIFWYTHHLFIVFYLLLLVHGMGGVIKHQ
TNLDAHTPGCISLNKTSSTNISLPEPEPEPEHAPNNTRWGTVKPCKEEPQFAPNPPQTWMWVVAPLVLYVVERLLRLLRKYQKVTIIKVVQHPCDVIEIRMKKKGFKAKPGQ
YVFINCPSISRFEWHPFTLTSCPSEKDDTFSVHIRIVGDWTERLADLLEEDSSEDSEHTQCRKFPELYIDGPFGSPSEDVFKYEVSICIAAGIGVTPFASILNSLRNKYIQD
DKPMKLRRLYFIWVCRDVQSFQWFADLLRELHRQLWEENRPDFLNIQLYLTGSDQKDAISSLNSRLHYGRPDWKQIFEEIAKEHPGTNVGVFYCGPRSLSKTLHKRCNRFNS
HKGTRFYYNKESFS
PP = 0.77

>Node 185 – Ancestral NOX123+NOX1234
MRSWLVNEGPRWLVLIIWLVINVVLFVYTFYNYQTSKEYYYLRVMLGYSLAFARGSAAVLNFNCALILLPVCRNLLSFLRGSCQVCSRNVRRLLDKNITFHKVIAWMICFHT
AIHVVAHLFNFERFVESYSSSNDSLLSRLSQLPNNGSDTWLNPIRYSNTDPHELFVKTVPGITGVIMTLVLILMVTSSTEAVRRSHFEVFWYTHHLFIIFFILLVVHGVGGV
IRRQTNLDEHNPPVCHNNKTRWGNPSDCPVPQFAPSGPTTWMWVIGPMVLYVLERLLRFRKSHQKVTIVKVVQHPSNVIEIQMKKKGFKAKPGQYVFINCPSISRFEWHPFT
LTSAPEDDYFSVHIRIVGDWTEKLAKLCGADEGEDQEARKFPEIYIDGPFGTASEDVFKYEVAILIGAGIGVTPFASILKSIWYKHNQDNSPMKLKKVYFFWICPDTQAFEW
FADLLQELEQQMQEQNMPDFLEIHIYLTGGWDENQARNIMLHEDDDVDAITGLRSRTHYGRPNWDKIFKKIAESHPNTNIGVFFCGPKALSKTLHKMCNKYSSAPDGTRFYY
NKENF
PP = 0.81

>Node 186 – Ancestral NOX123
MRSWLVNEGPRWLVLIIWLVINVILFVYTFFNYQTSKEYYYLRVLLKSALAFARGSAAVLNFNCALILLPVCRNLLSFIRGSCQVCSRNIRRLLDKNITFHKAIAWMICLHT
AIHVVAHLFNFERFVESYSSSNDELLSRLSQLPNNGSDTWLNPIRYSNTDPHELFVKTVPGITGVIITLVLILMITSSTELIRRSYFEVFWYTHHLFIIFFIGLVVHGVGGI
IRRQTNLDEHNPPVCHNKHSRWGNPSDCPVPQFAPSGPTTWMWVIGPMVLYVLERLLRFRKSHQKVVITKVVQHPSNVIEIQMKKKGFKAEPGQYVFINCPSISRFEWHPFT
LTSAPSDDYFSVHIRIVGDWTEALAKACGADEGEFQEAWKMPEIYIDGPFGTASEDVFKYEVAILIGAGIGVTPFASILKSIWYKYNQDNQGLKLKKVYFFWICPDTNAFEW
FADLLQELEQQMVEQGMADFLEYHIYLTRGWDENQARNIMLHEDDEVDAITGLRSKTHYGRPNWDKIFKDIAESHPGTNIGVFFCGPKALSKTLHKMCNKYSSAPDGTRFYY
NKENF
PP = 0.83

>Node 205 – Ancestral RBOH
MQPLSNRHTSSSETFEGKDAAGESGPGTGGKLLPIFRNPASRNSGNSSGNADEEFVEVTLDLRDDSVILQEIESASAGPSAASPELTLLEKNLSKNSTLGASIIRSSSVRLR
QVSQELKRLRSFNARAGRKQRAKLDRTKSSAARALRGLRFISKTTTGDGWREVEKRFDQLAADGLLPREKFGECIGMKESKEFAAELFDALARRRRIKTENINKDELREFWE
QITDQSFDARLQIFFDMVDKNGDGRITEEEVKEIIMLSASANKLSKLKEQADEYAALIMEELDPDNLGYIELWQLETLLRQMPTHSVNLTNESRKTSQTLSQNLKPTRERNP
IKRCYCKLKYLLQDNWKRIWVLTLWIMINVGLFIWKFLQYKRKAAFQVMGYCVCVAKGAAETLKFNMALILLPVCRNTLTWLRSTRLGRIVPFDDNINFHKVIAWAIAIGTL
LHAVSHLACNFPRLTHASEEEFQTLSSYFGNTQPKTYLNLVKSVPGITGIIMVVLMAIAFTLATPWFRRNRIKLPKPFNRLTGFNAFWYSHHLFVIVYILLIIHGYFLYLTK
KWYKKTTWMYLAVPVILYACERLLRVFRSHYYSVKILKVAVYPGNVLALHMSKPPGFKYKSGQYMFVNCPAISPFEWHPFSITSAPGDDYLSVHIRTLGDWTQELKRVFSEA
CEPPTAGKSGLLRADTKESKKNFPEIFIDGPYGAPAQDYKKYDVLLLVGLGIGATPFISILKDLLNNIKEQEQGADSGNSDISNGTSESPKTKKSTLRTRRAYFYWVTREQG
SFDWFKGVMNEVAEMDHKGVIEMHNYLTSVYEEGDARSALITMVQSLHHAKNGVDIVSGTRVRTHFARPNWKKVFSRIATKHPNSRIGVFYCGAPALTKELRKLCQEFSQKT
STRFEFHKENF
PP = 0.8

>Node216 – Ancestral DUOX
MLRFLVCVLLLVLSTGTLGNEPTTEETIFSHNVEYQRYDGWYNNLAHPDWGSVGSPLTRRVPPAYADGVYQPAGSNRPNPRTISNALFRGPTGLPSTRNRTTLFAFFGQLVA
YEILQASEPGCPAEILNIEIPECDPTFDKDCRGEKFMPFLRSRYDKNTGQSPNNPREQLNEVTSWIDGSFIYSTSEAWANALRSFKGGRLASDDGSNFPPYNTIGLPLANPP
PPATHKLRDPERLFKLGDPRSNENPALLAFGILFFRWHNYLADRIQRQHPDWSDEKIFQRARRWVIATLQNIVLYEWLPAFLGEEVPPYTGYKPHIHPSISHVFQAAAMRFG
HTMVPPGIYLRNAQCHFRKTVAGYPALRLCNTYWNSQDVLQETGIEELLMGMASQIAEREDNVIVEDLRGHVFGPLHFSRRDLVALNIMRGRDHGLPDYNTARKAFGLPPIT
NWQDINPDLYKTNPEILERLKELYNNDINKIDIYPGGLLETTSNGPGELFTTIIKDQFLRIRDGDRFWFENRQNGLFTEEEIEEIRNITLYDVIIAATNINPNQIQKNVFFW
REGDPCPQPHQLNESDLEPCTPLHGYDYFSGSEVSYIFTFIALGLIPLVCVLVAYIILARRRKRKLNKARKRTRQNVRKEKKESTDEFHALEWLGHKEHSRPVQVKLGPDATI
RVTNRKGEVLRTIDLKHLQAVTIFVSANKNRPFMLLRIPKEYDLVLRFDNEEERNKFINELEDFLKGHGKGVEIREMKQKELLTSAETKEKRQKLLERFFREAFAQAFNLDE
DEDSKQLDNSEDCKDVLDCELSKEEFAEALGMKPNSLFVEQMFSLVDKDHNGYISFREFLDVVVLFAKGSAEDKLKLLFNMYDVDGNGQISREELKEMLKSLMEMANSSLSE
EQIDELVNSMFKSAGLEDKEELTFEDFKVLLKDHKEELSNVSLDLKGVNQNEQTRRQNVPSRARKTIVKAYAERRQTRERVETVKSKYQSTPLKAKWNRFKRFLENYRQHIF
YLVLFYAICLGLFVERFYYYSFESEHAGLRRIMGYGIAITRGAAAAMSFTFSLLLLTMCRNLITWLRETFLNRYIPFDSAISFHKIVAWTALFFTVIHTIGHCINFYHVSTQ
PLSHLKCLFRDIFFRSDYLPKFHYWLFQTITGLTGVLLVLVMCIMYVFATPTVRRHAFNAFWLTHHLYILLYILTILHGSARLVKKPTFWYYFIGPAIIFVLDKLISLKKKY
KELSVIKAELLPSDVTYIEFKRPPNFEYKSGQWVRISCPALGSNEYHPFTLTSAPHEDTLSVHIRAVGPWTWKLRNIFDPNNVKDQPYPTLYLDGPYGAGNQDWYKYEVAVL
VGAGIGVTPYASILKDLVHRTSTNKHSRIKCKKVYFIWVTRSQKHFEWFVDILREVEEKDTTDLLEVHIFITQFFQKFDLRTTMLYICEKHFQKLSGRSMFTGLKAITHFGR
PDFESFFESVQEKHPQVSKVGVFSCGPPGLTKSVEKACQEVNKQEKAHFIHHFENF
PP = 0.78

>Node 239 – Ancestral NOXC
MAENAVSLELVPPSYADGVGLPSLRNRTTLFVYFGEILPATGPGCPPDGRGGRLLPLLRTRHDKNTSLSPIYSTSEAWADLLRSFKGGKLASDEGLEFPPENSIGLPTANPP
PPATAKLRGNENPAVRASRPLGLLFYRYHNALAEAIARAHPDRDRRWTIATLQKIILHEWLPAFLGHLQPSTSTLVPPGTLTRSGACHFRGTLLKSPALRLCGLPDYPPLTN
WEDINPLLHDDDLNDLDLFPGGLLETEADPPGPEELKDVLDAQLDREEFAEALGMKPSSLFAERLFSLVDKDRTGSISFKELLDALNMLTHGSSDDKLKLLFDMYDTDGDGR
IDYEELKQVLRSAMEANGLSLSPEQLDELVPKPSAHHVLHTDHDNDARNAILKSLDADGDGKISFEDFKLLLRKYPEELANLSLSAASLKAYEERKKSRTLTESLKPEEEKS
RVRTPWRRLRRYLQNHGPKIIFLVLFLLINLGLFVWSFFQYKNSPEYSLRKIMGYSVAIARGCAAVLNFNCAFILLPMCRNLLTWLRSTRLNRYIPFDKSITFHKLIGWVIV
FFSLVHTVCHLCFNFSHLAEDPAAVYYADALPPKFAYLLFVKSIPGLTGWILVVVLVIMFVTSTPAVRRSGFEVFWYTHHLFIPIIVLLILHGSGALIKPPTFWYYLVVPAV
LYVLERLLRVKCFRLKVTIVKVLLPSDTVELVMKRPPGFKYKPGQYVFINVPAISRFQWHPFTITSCPSEEDYLSVHIKAVGDWTGKLYELFDENEDEEKPLLGDADDNALV
EPPPGGKSGTARKLPTKPTALLKLVNIEVYIDGPYGAPSQDFFKYEHAVLIGAGIGVTPFASILKDLVHRHKSGRQSCPSANTDWSDDTSEEPPKRDESGDGAPGSKMKLRR
VDFFWITRDQKSFEWFADLLRELEEEQAEEGNLDDFLEIHISLTSARGKSDLKSVMLHMALELHAHKKNGRSLITGLRTSRTHYGRPDWDKIFEKIREEVLKLRAEEHPNGS
KVGVFYCGPPALAKTLRKACRKYNKKERKGEGGTRFEFHKENF
PP = 0.57

>Node 243 – Ancestral NOX5
MTTARENNEWGSVGSPLSLEVPPSYSDGPYKPAPAQQPNPRSASNLSRGAANLPEKRTRGDKNTSLLDRLGSPKGNENPAIRLFGLLFYRYNNCLAELVAREVFSKDRRLTI
ATLQKVILHEPSSSQLFQLTQAEIIGLASQLTENDDQNIEHDLEGKGFLERFEFLKGKGKGVELKEVGSLELLLSADEDEKWLKWIEKRFKEVAGEDRQIDLEEFKKALGMK
KSFFAERLFSLFDKDRSGSISLNELLDALNMLTHGSSTDKLKFLFDMYDVDGNGLIDHEELRTVLRSCMEENALSLSPEQLDELTNALFESADADGSGTISFEEFKVLLEKY
PEVLENLTISAASWLKPPEKKRKKNRKRTPYYRLWRYLRNNRKQVVFLVLFLLINIGLFVWAFYQYRDSYSVAIARGCGQCLNFNCAFILIFMLRKCLTWLRSTRLGRYLPL
DQSITFHKLIGWVIVFFSLVHTVCHLLNFSSLAENPSNNFTYWEYLFPTKTGIGWVGGIASLTGWILVVIILIIMFVTSLPFVRRSHYFEVFYYTHLLYIPFLVLLILHGPNF
WYFFIVPAVIYILERILRVKCIRLARYGKTTIKEVNLLPSNVVELVMKRPPGFKYRPGQYVFINIPAISKFEWHPFTISSCPEQQDTLSLHIRAVGNWTKRLYEYFEEKEKK
EKALAEKSPTKSTLSRKTRRPGKLGTLLREVHVEVYIDGPYGAPSQDFFKYEHAVLIGAGIGVTPFASILQSLMHRHRSGRQTCPKCNHSWTDDVPGSKMKLKKVDFFWITR
DQKSFEWFADLLQELEEEQAEEGNLDDFLDMHIYLTSARGKSDLKSVGLQMALDLLHKKHNRDLITGLRTRTQYGRPDWDEIFEKIVEEKGGKVGVFYCGPPALAKTLRKAC
QKYNFEFHKENF
PP = 0.63
